# Neutrophils to lymphocytes ratio and platelets to lymphocytes ratio in pregnancy: A population study

**DOI:** 10.1371/journal.pone.0196706

**Published:** 2018-05-22

**Authors:** Anat Hershko Klement, Efrat Hadi, Aula Asali, Tal Shavit, Amir Wiser, Einat Haikin, Yael Barkan, Tal Biron-Shental, Alona Zer, Yifat Gadot

**Affiliations:** 1 Obstetrics and Gynecology Division, Meir Medical Center, Kfar Saba, Israel and Tel Aviv University, Tel Aviv, Israel; 2 Clalit Health Services, Netanya, Israel; 3 The Institute for Oncology, Rabin Medical Center, Tel Aviv University, Tel Aviv, Israel; 4 Obstetrics and Gynecology Division, Kaplan Medical Center, The Hebrew University of Jerusalem, Jerusalem, Israel; University of Toronto, CANADA

## Abstract

**Background:**

Neutrophils to lymphocytes ratio (NLR) and platelets to lymphocytes ratio (PLR) are both inflammatory ratios that can be easily calculated from a simple blood count. They are frequently reported and tested as prognostic factors in several medical disciplines. Pregnancy involves special reference values for laboratory assays.

**Objective:**

The aim of this study was to define pregnancy-related reference values for NLR and PLR according to trimester, background morbidity and according to the patient's age.

**Study design:**

A retrospective analysis of a large cohort undergoing community-based pregnancy surveillance between the years 2011–2016. Data were analyzed according to high-risk patient versus normal-risk patient.

**Results:**

A total of 11,415 patients were included. Mean PLR and NLR values were 136.3±44.3, 2.6±1, respectively during the first trimester, 144.6±47.1, 4.0±1.4 respectively during the second trimester and 118.1±42.0, 3.5±1.2 respectively during the third trimester. No difference was detected between the high-risk and the normal population (P-values 0.3, 0.5 and 0.4 for PLR in each trimester respectively and 0.3, 0.4, 0.6 for NLR in each trimester, respectively). No differences were detected among parity categories. The correlation between patient’s age and either PLR and NLR was a weak positive correlation (though statistically significant). Both PLR and NLR reached a maximum value during the second trimester. The differences between mean NLR and PLR between trimesters were significant (P <0.01 for all differences tested). PLR rises in the presence of anemia, reaching statistical significance (P-value for PLR in each trimester was <0.01). NLR showed an opposite trend (P-values for NLR were 0.4, 0.005 and 0.06 in each trimester, respectively).

**Conclusions:**

In our cohort, there were generally no differences between the high-risk and the normal population, excluding patients with a fibroid uterus or inflammatory bowel disease who presented a significantly elevated PLR through all trimesters. Both PLR and NLR reached a maximum value during the second trimester and were positively correlated with age. We anticipate that the population-based data will assist in providing accurate reference values for future research testing NLR and PLR measures during pregnancy.

## Introduction

Neutrophils to lymphocytes ratio (NLR) and platelets to lymphocytes ratio (PLR) are both inflammatory ratios that can be easily calculated from a simple blood count. Due to the fundamental meaning of these ratios, reflecting an inflammatory load, they are frequently reported and tested as prognostic factors in several medical disciplines. Numerous publications (only a few recent articles are quoted) investigated these measures in cardiology [1], oncology [2], surgery [3], and gastroenterology [4], often incorporating them into the prognostic algorithm [5–8]. In the gynecology-related literature, NLR and PLR were evaluated in gynecological cancers [9] and in reproductive morbidities such as ovarian hyperstimulation syndrome [10], premature ovarian insufficiency [11] and endometriosis [12]. In obstetrics, NLR was reported to be increased in patients with hyperemesis gravidarum [13], gestational diabetes [14], pre-eclampsia [15], pregnancy-associated intrahepatic cholestasis [16] and other diseases. The interpretation of elevated values in pregnancy is far from trivial and is intriguing. Pregnancy involves many physiological changes, resulting in special reference values for laboratory assays.

The aim of the present study was to define pregnancy-related reference values for NLR and PLR according to trimester and patient age in a normal-risk population.

## Materials and methods

This is a retrospective analysis of patients undergoing community-based pregnancy surveillance through Clalit Health Services Health Maintenance Organization (CHS HMO) from 2011 through 2016. Patients were routinely referred for an automated blood count in each trimester of pregnancy by their primary care physician in the ambulatory setting. The first trimester was defined as 4–14 weeks from the last menstrual period (LMP), the second trimester as 14.1–28 weeks from LMP, and the third trimester as 28.1–40 weeks from LMP. Dating was validated by an ultrasound crown to rump (CRL) measurement performed before 14 weeks of gestation. In our health care system, the first routine surveillance visit is scheduled for the seventh week of gestation and the last visit for 36–37 weeks. Routine blood samples are taken once in each trimester; first sample at 7.1–14 weeks, second at 14.1–28 weeks, and the third at 28.1–37 weeks of gestation. The compliance rate for routine pregnancy-related laboratory follow-up for the study population was monitored by the CHS HMO. In a survey of 6,308 pregnant patients, 5,395 had the prescribed blood tests (85.5% compliance rate, unpublished data). Supervision of pregnancies beyond 37 weeks is performed by hospitals. As a general health policy, all patients beyond 40 weeks of gestation are referred to a secondary or tertiary medical center for post-date surveillance.

All blood counts were performed by a single lab, using the same automatic computerized technology (ADVIA 2120i Hematology System with Autoslide, Siemens). Of note, is that all patients were prescribed an iron supplement at the first pregnancy surveillance visit.

Data collected included information on maternal age, parity, designation as low-risk or high-risk pregnancy (HRP), sociodemographic level of the primary clinic (as defined by the national Ministry of Health) and blood count parameters from samples taken from each patient at each trimester of the pregnancy including hemoglobin concentration (g/dL), platelet count (K/microL), and neutrophil count (K/microL). The raw data used in this study are included in supplementary files S1 File, S2 File, and S3 File.

High-risk classification in the study population involved a community-based fetal-maternal medicine clinic supervised by a fetal-maternal specialist. A HRP was defined as one involving a pre-existing medical condition (including all morbidities related to the cardiovascular, pulmonary, hematology, gastroenterology, hematology, endocrinology, or neurology systems, as well the presence of an infectious disease, mental disease, obesity (body mass index during the first trimester >30), drug addiction or alcohol abuse. Patients presenting with the following obstetrical history were also defined as high-risk: recurrent pregnancy loss, preterm delivery, small for gestational age fetus, fetal or neonatal malformation, gestational diabetes, pregnancy-related hypertensive disorder, intrauterine fetal death, uterine anomaly, three or more low-transverse cesarean deliveries and corporal cesarean delivery. Patients classified as high-risk in the current pregnancy included those presenting with multiple fetal gestation, fetal anomaly, fetal growth restriction, positive indirect coombs test, cervical shortening, documented exposure to an infectious disease that can be transmitted to the fetus, elevated blood pressure and gestational diabetes.

Data were analyzed according to high-risk versus normal-risk patients. Sub-analysis of the various background morbidities was performed for the HRP when the diagnosis was included in the electronic medical record.

### Statistical analysis

PLR, NLR and hemoglobin (Hb) were described using arithmetic mean and standard deviation for each trimester. In addition, each trimester was divided into 7 percentiles (3rd, 10th, 25th, 50th, 75th, 90th and 97th) and plotted on a separate line for each parameter. Error bars for the plots were calculated based on one standard deviation from the mean. Differences between categories in study parameters were tested for significance using the *t-test* for independent samples. Differences between trimesters were tested for significance using ANOVA for repeated measurements. P-values were adjusted with Bonferroni method. For maternal age association, the samples were divided into year intervals, while patients ≤24 years and ≥45 years were grouped into lower and upper groups. Age plots show mean parameters by age and pregnancy trimester, while error bars represent one standard deviation from the mean. All calculations were performed using R stats software (R Core Team 2013. R: A language and environment for statistical computing. R Foundation for Statistical Computing, Vienna, Austria. ISBN 3-900051-07-0, URL http://www.R-project.org/).

The study was approved by the local ethics committee (Number MMC0074-16-COM1). The committee did not require informed consent because all patient data were retrospective and fully anonymized.

## Results

A total of 11,415 consecutive patients were included, although some did not have a blood count in each trimester (11,413 in trimester 1, 11,388 in trimester 2, and 11,415 in trimester 3). The mean NLR and PLR are presented in Tables 1 and 2, respectively. No difference was detected between the high-risk and the normal-risk populations (P = 0.3, 0.5 and 0.4 for PLR in each trimester, respectively and 0.3, 0.4, 0.6 for NLR in each trimester, respectively). No differences were detected among parity categories. Interestingly, both PLR and NLR reached a maximum value during the second trimester. The differences between mean NLR and PLR between trimesters were significant (P <0.01 for all differences tested, adjusted by Bonferroni method).

**Table 1:** Mean platelets to lymphocytes ratio (PLR) values by trimester and high-risk pregnancy (HRP) condition.

Trimester	HRP	Number of patients	Mean	SD^a^
Trimester I	No	6235	135.87	44.81
	Yes	5178	136.76	43.59
	Total	11413	136.27	44.26
Trimester II	No	6224	144.37	46.71
	Yes	5164	144.99	47.52
	Total	11388	144.65	47.08
Trimester III	No	6242	117.84	41.30
	Yes	5173	118.52	42.91
	Total	11415	118.15	42.04

^a^SD = standard deviation

**Table 2:** Mean neutrophils to lymphocytes ratio (NLR) values by trimester and high-risk pregnancy (HRP).

Trimester	HRP	Number of patients	Mean	SD^a^
Trimester I	No	6235	2.60	1.01
	Yes	5178	2.62	0.99
	Total	11413	2.61	1.00
Trimester II	No	6224	4.04	1.34
	Yes	5164	4.06	1.37
	Total	11388	4.05	1.36
Trimester III	No	6242	3.48	1.19
	Yes	5173	3.49	1.20
	Total	11415	3.48	1.19

^a^SD = standard deviation

**Fig 1:**
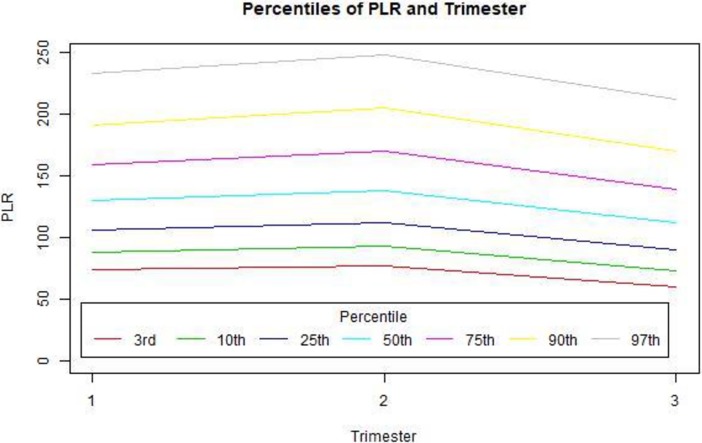
PLR percentiles by trimester.

**Fig 2:**
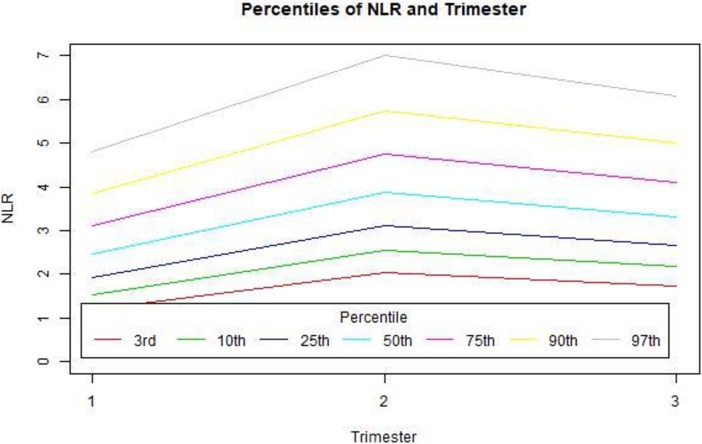
NLR percentiles by trimester.

**Fig 3:**
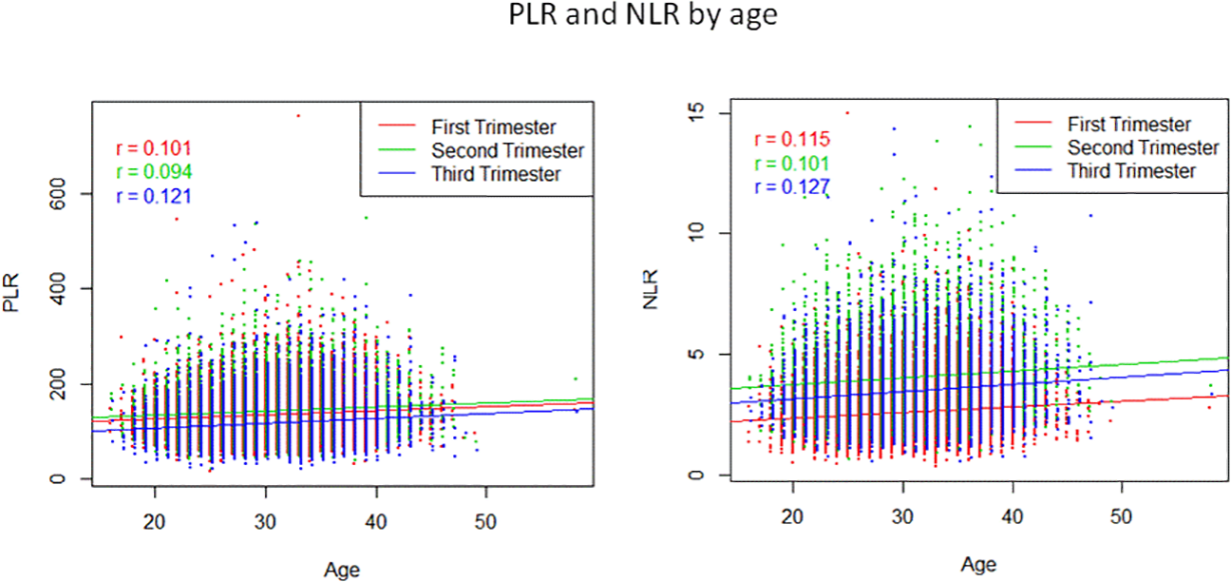
PLR and NLR by age.

As no difference was demonstrated between the high-risk and normal-risk pregnancy groups, we combined all data and pooled the values into detailed percentile presentations (Tables 3 and 4, Figs 1 and 2). The correlation between patient’s age and either PLR and NLR was a weak positive correlation (though statistically significant) and was more pronounced in the third trimester for both measures (Fig 3).

**Table 3:** PLR percentiles by trimester.

Trimester	3%	10%	25%	50%	75%	90%	97%
Trimester I	73.9	87.7	106.2	130	158.6	190.7	232.3
Trimester II	76.8	93	112.3	137.6	169.5	204.6	247.1
Trimester III	59.6	72.9	89.5	111.7	138.9	169.4	211.9

**Table 4:** NLR percentiles by trimester.

Trimester	3%	10%	25%	50%	75%	90%	97%
Trimester I	1.2	1.5	1.9	2.5	3.1	3.8	4.8
Trimester II	2	2.5	3.1	3.9	4.8	5.8	7
Trimester III	1.7	2.2	2.7	3.3	4.1	5	6.1

**Fig 4:**
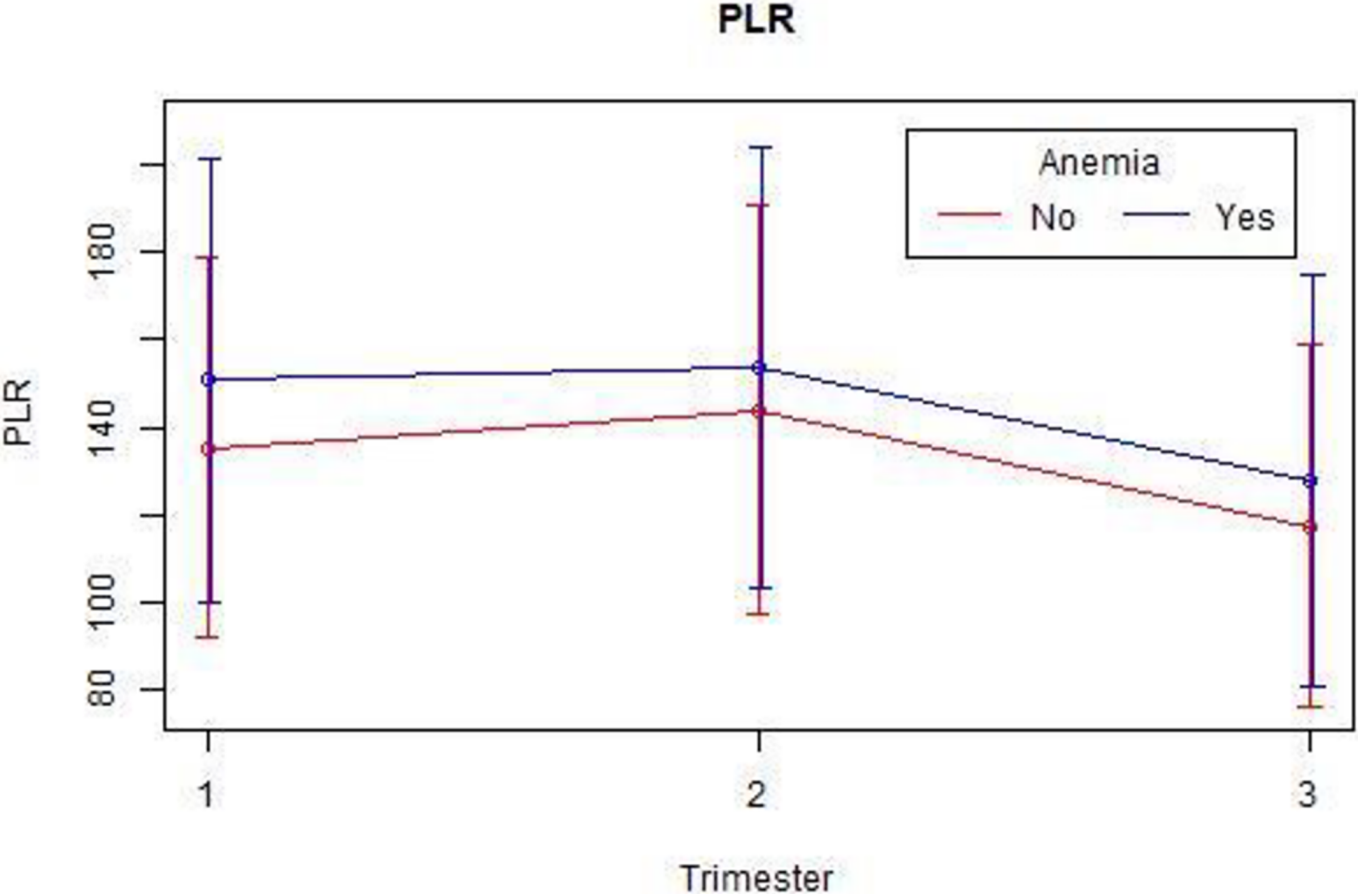
PLR mean values by trimester and anemia.

**Fig 5:**
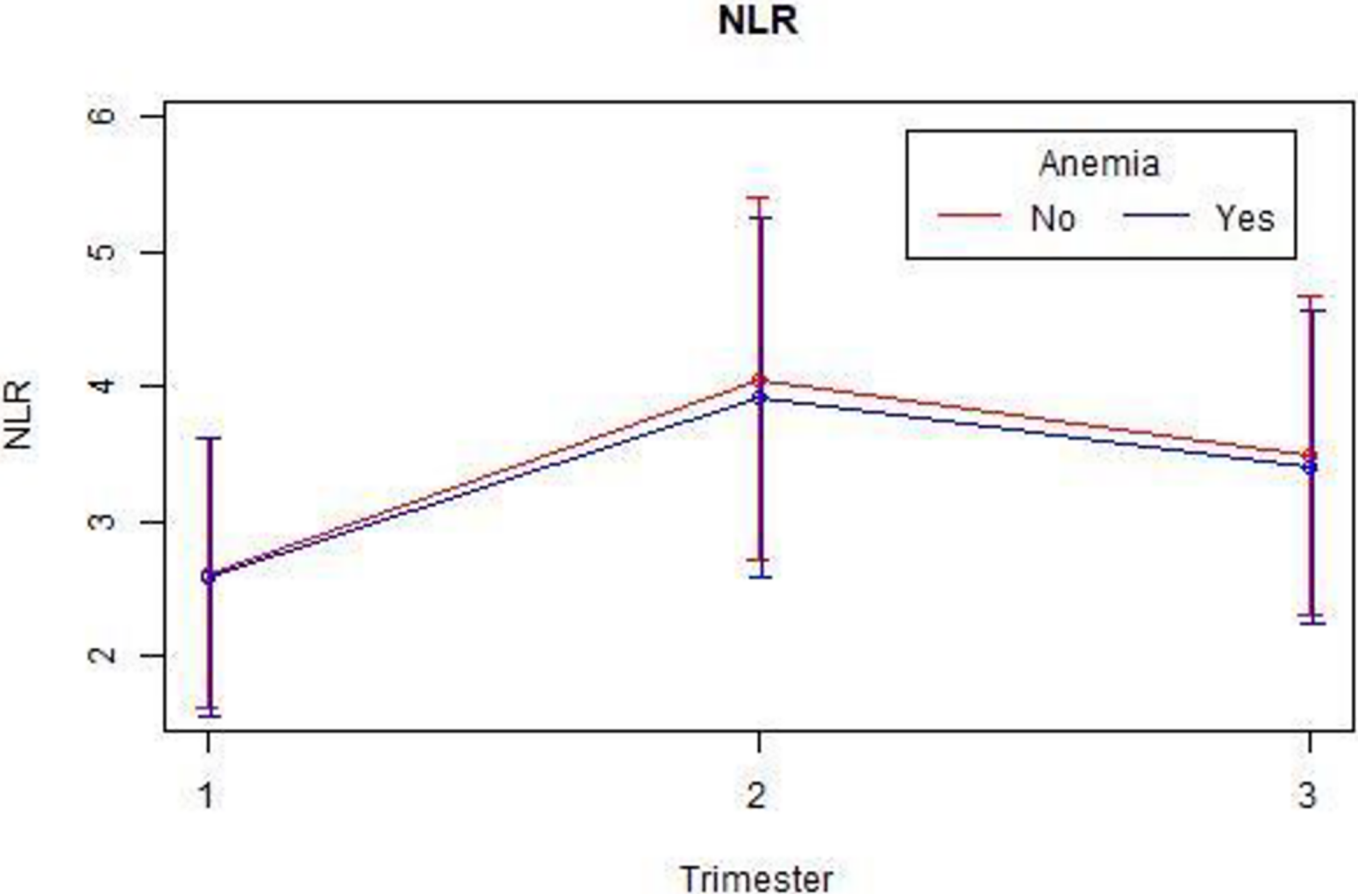
NLR mean values by trimester and anemia.

We also extracted the hemoglobin data from each trimester (Table 5) and tested whether anemia (defined in pregnancy as hemoglobin <11 g/dL) affects the NLR and PLR values. As expected, the PLR increases in the presence of anemia (Table 6, Fig 4), reflecting a known platelet reaction in the presence of anemia and reaching statistical significance (P-value for PLR in each trimester was <0.01). NLR showed an opposite trend (Table 6, Fig 5); Although not consistently significant, P-values for NLR were 0.4, 0.005 and 0.06 in each trimester, respectively. For future studies, we provide age-based reference tables and figures for each of the above measures (Tables S1 Table, S2 Table and S3 Table, Figures S1 Fig, S2 Fig and S3 Fig).

**Table 5:** Mean hemoglobin (HGB) values (g/dL) by trimester and high-risk pregnancy (HRP) status.

Trimester	HRP	Number of patients	Mean HGB	SD^a^
Trimester I	No	6235	12.26	0.88
	Yes	5178	12.27	0.89
	Total	11413	12.26	0.88
Trimester II	No	6224	11.13	0.87
	Yes	5164	11.13	0.87
	Total	11388	11.13	0.87
Trimester III	No	6242	11.44	1.00
	Yes	5173	11.44	1.00
	Total	11415	11.44	1.00

^a^SD = standard deviation

**Table 6:** Mean PLR and NLR by trimester and gestational anemia.

Trimester	Anemia	Number of patients	Mean PLR	SD^a^ PLR	Mean NLR	SD^a^ NLR
Trimester I	No	10649	135.24	43.55	2.61	1.00
	Yes	764	150.72	51.05	2.58	1.03
	Total	11413	136.27	44.26	2.61	1.00
Trimester II	No	10605	143.97	46.76	4.06	1.36
	Yes	763	153.78	50.65	3.92	1.34
	Total	11368	144.63	47.09	4.05	1.36
Trimester III	No	10633	117.43	41.54	3.49	1.19
	Yes	763	127.98	47.20	3.41	1.17
	Total	11396	118.13	42.03	3.48	1.19

^a^SD = standard deviation

Since approximately 2,600 cases classified as HRP were further identified by reason for the special supervision, we tested the characteristics of these sub-groups (S4 Table). Statistically significant elevated PLR through all trimesters was demonstrated for women with fibroid uterus and for patients diagnosed with an inflammatory bowel disease. NLR was not consistently statistically elevated for any of the sub-groups. The socioeconomic analysis (S5 Table) demonstrated a trend for a higher PLR and a lower NLR in the case of the lower class. The values showed a statistically significant difference (P<0.05) in the case of PLR during the third trimester and for NLR during the first and second trimesters.

## Discussion

To the best of our knowledge, this is the first study to compare NLR and PLR values during pregnancy in a low-risk population versus a high-risk population. The importance of these nomograms arises from the ability to properly interpret future values in studies evaluating PLR and NLR ratios during pregnancy. In our cohort, there was generally no difference between the high-risk and the normal-risk populations. Subtle changes were detected in specific morbidities, such as fibroid uterus and inflammatory bowel disease. Both PLR and NLR reached a maximum value during the second trimester and were positively correlated with age.

NLR and PLR were previously tested as predictors of common pregnancy complications, but the results were inconsistent [14, 15, 17, 18]. For example, NLR was not found to be predictive for pregnancy induced hypertension, [18] while others found it to be significantly increased in patients with pre-eclampsia [15]. Similar contradictions were reported in studies testing NLR and PLR in parturients with gestational diabetes. NLR and PLR were retrospectively analyzed in healthy and pregnant women with gestational diabetes and were not found to be predictive [14]. Others reported NLR and PLR were significantly higher in gestational diabetes compared with a control group [17]. We presume that some of the discrepancy can be settled by a well-established reference value system for each trimester.

NLR was also assessed in inflammatory conditions complicating pregnancy and was suggested as an early marker of acute pancreatitis in pregnancy and a possible marker for disease severity [19]. A more common condition is placental inflammatory response, where NLR was shown to have better diagnostic performance than maternal serum CRP. High NLR was predictive of impending preterm delivery in the context of normal CRP levels [20]. These reports emphasize the potential added value of evaluating the NLR in diagnostic challenges during pregnancy.

Although the current study was retrospective, it is based on a large sample, using standardized sampling, as all blood samples from ambulatory settings were analyzed by a single lab. In addition, although we could accurately define the patients with no risk factors, the database was partially able to stratify for specific risk factors in the high-risk population. Therefore, some subgroups might still have elevated NLR and PLR as part of the background pathophysiology. In addition, certain acute conditions could not be included, because the study took place in a primary care setting and the diagnosis of high-risk pregnancy in our specific primary care setting does not encompass all obstetrical morbidities.

To the best of our knowledge, this is the largest cohort analysed for neutrophils to lymphocytes ratio (NLR) and platelets to lymphocytes ratio (PLR) in pregnancy. Since these ratios are of value in general medicine, further testing of their prognostic value is required in pregnancy-related morbidities. We anticipate that the nomograms provided by the analysis presented here will serve future researchers and will assist in providing an accurate assessment of the utility of these measures.

## Supporting information

S1 File HRP analysis codes. (XLSX)

S2 File Dataset. (CSV)

S3 File HRP diagnosis. (XLSX)

S1 Table Mean PLR by age and pregnancy trimster. (DOCX)

S2 Table Mean NLR by age and pregnancy trimester. (DOCX)

S3 Table Mean hemoglobin by age and pregnancy trimester. (DOCX)

S4 Table Mean PLR and NLR by specific diagnosis and trimester (referral group: uncomplicated pregnancies). (DOCX)

S5 Table Mean PLR and NLR by socioeconomic class. (DOCX)

S1 Fig PLR age plots. Mean parameters by age and trimester (error bars represent one standard deviation from the mean).
(DOCX)

S2 Fig NLR age plots. Mean parameters by age and trimester (error bars represent a single standard deviation from the mean).
(DOCX)

S3 Fig Hemoglobin age plots. Mean parameters by age and trimester (error bars represent a single standard deviation from the mean).
(DOCX)
