# Supplementary material for: Neutrophils to lymphocytes ratio and platelets to lymphocytes ratio in pregnancy: A population study
Source: PLoS One. 2018 May 22;13(5):e0196706. doi: 10.1371/journal.pone.0196706 (PMC5963784; doi:10.1371/journal.pone.0196706)
Supplement: S1 Table — (DOCX) [file pone.0196706.s004.docx]

S4Table. Mean PLR by age and pregnancy trimester

| **Age** | **N** | **Trimester I Mean (SD^a^)** | **Trimester II Mean (SD^a^)** | **Trimester III Mean (SD^a^)** |
| --- | --- | --- | --- | --- |
| <24 | 1304 | 130 (41.3) | 138.4 (44.7) | 112.2 (40.5) |
| 25 | 478 | 129.9 (40.3) | 135.3 (39.8) | 110 (38.5) |
| 26 | 486 | 132.8 (42.7) | 141 (43.3) | 111.5 (36) |
| 27 | 626 | 132.4 (43.2) | 141.5 (43.9) | 115.2 (43.3) |
| 28 | 722 | 130.9 (41.6) | 145.7 (49.7) | 118 (41.9) |
| 29 | 842 | 132.8 (41.6) | 143.2 (49.1) | 114.1 (43.6) |
| 30 | 869 | 133.7 (45.4) | 142.2 (44.7) | 114.4 (40.3) |
| 31 | 869 | 135.3 (41.9) | 144.5 (46.3) | 116.9 (40.6) |
| 32 | 905 | 137.6 (44.6) | 143.4 (46.5) | 117.1 (41.2) |
| 33 | 798 | 141.1 (54.7) | 144.9 (47.8) | 120.6 (41.4) |
| 34 | 734 | 141.2 (45.9) | 147.8 (49.3) | 119.9 (40.7) |
| 35 | 663 | 141.9 (41.4) | 150.2 (45.7) | 124.2 (41.8) |
| 36 | 552 | 138.5 (42.3) | 146 (45) | 123.2 (41.5) |
| 37 | 444 | 143 (44.3) | 151.6 (57.3) | 123.7 (46) |
| 38 | 373 | 143.2 (44.3) | 152.3 (47.6) | 129.1 (45.2) |
| 39 | 300 | 143.3 (45.9) | 151.8 (53.6) | 125.7 (45.2) |
| 40 | 184 | 149.3 (50.3) | 156.1 (42.1) | 130.3 (37.7) |
| 41 | 115 | 143.8 (44.7) | 153 (45.4) | 133.8 (49.3) |
| 42 | 59 | 145.7 (39.6) | 158.5 (44.9) | 136.5 (43.4) |
| 43 | 41 | 142.5 (45.2) | 160.8 (61.2) | 142.5 (67.6) |
| 44 | 14 | 146.1 (45.7) | 154 (38) | 130.4 (19.2) |
| >45 | 35 | 129.6 (31.6) | 157.3 (52.9) | 127.9 (50.3) |

^a^SD=standard deviation
