# Supplementary material for: Neutrophils to lymphocytes ratio and platelets to lymphocytes ratio in pregnancy: A population study
Source: PLoS One. 2018 May 22;13(5):e0196706. doi: 10.1371/journal.pone.0196706 (PMC5963784; doi:10.1371/journal.pone.0196706)
Supplement: S3 Table — (DOCX) [file pone.0196706.s006.docx]

## S8 Table. Mean hemoglobin by age and pregnancy trimester

| **Age** | **N** | **Trimester I Mean (SD**^a^**)** | **Trimester II Mean (SD**^a^**)** | **Trimester III Mean (SD**^a^**)** |
| --- | --- | --- | --- | --- |
| <24 | 1304 | 12.2 (0.9) | 11 (0.9) | 11.3 (1.1) |
| 25 | 478 | 12.3 (0.9) | 11.1 (0.9) | 11.4 (1) |
| 26 | 486 | 12.3 (0.9) | 11.1 (0.8) | 11.5 (1) |
| 27 | 626 | 12.4 (0.9) | 11.2 (0.9) | 11.5 (1) |
| 28 | 722 | 12.3 (0.9) | 11.2 (0.9) | 11.5 (1.1) |
| 29 | 842 | 12.3 (0.9) | 11.2 (0.9) | 11.5 (1) |
| 30 | 869 | 12.3 (0.9) | 11.2 (0.9) | 11.5 (1) |
| 31 | 869 | 12.4 (0.9) | 11.2 (0.9) | 11.5 (1) |
| 32 | 905 | 12.3 (0.9) | 11.1 (0.8) | 11.4 (1) |
| 33 | 798 | 12.2 (0.9) | 11.2 (0.9) | 11.4 (1) |
| 34 | 734 | 12.2 (0.9) | 11.1 (0.8) | 11.4 (0.9) |
| 35 | 663 | 12.2 (0.9) | 11.1 (0.9) | 11.4 (1) |
| 36 | 552 | 12.2 (0.8) | 11.1 (0.8) | 11.4 (0.9) |
| 37 | 444 | 12.2 (0.9) | 11.1 (0.9) | 11.4 (0.9) |
| 38 | 373 | 12.2 (0.9) | 11 (0.8) | 11.3 (0.9) |
| 39 | 300 | 12.2 (0.8) | 11.1 (0.8) | 11.4 (0.9) |
| 40 | 184 | 12.1 (0.9) | 11 (0.7) | 11.4 (0.9) |
| 41 | 115 | 12.2 (0.8) | 11 (0.9) | 11.4 (1) |
| 42 | 59 | 12.1 (0.8) | 10.8 (1) | 11 (0.9) |
| 43 | 41 | 12.2 (1) | 11 (0.9) | 11.4 (1) |
| 44 | 14 | 12.1 (1.2) | 10.9 (0.7) | 11.6 (0.9) |
| >45 | 35 | 12.5 (0.7) | 11.2 (0.9) | 11.6 (0.9) |

^a^SD=standard deviation
