# Supplementary material for: Neutrophils to lymphocytes ratio and platelets to lymphocytes ratio in pregnancy: A population study
Source: PLoS One. 2018 May 22;13(5):e0196706. doi: 10.1371/journal.pone.0196706 (PMC5963784; doi:10.1371/journal.pone.0196706)
Supplement: S4 Table — (DOCX) [file pone.0196706.s007.docx]

S10 Table. Mean PLR and NLR by specific diagnosis and trimester (reference group: uncomplicated pregnancies)

PLR

| **Diagnosis** | **Number of patients** | **Mean Trimester 1** | **SD^a^** | **P-value** | **Mean Trimester 2** | **SD^a^** | **P-value** | **Mean Trimester 3** | **SD^a^** | **P-value** |
| --- | --- | --- | --- | --- | --- | --- | --- | --- | --- | --- |
| Hyperemesis gravidarum | 413 | 136.50 | 39.57 | 0.757 | 148.50 | 47.62 | 0.08853 | 118.27 | 40.72 | 0.835 |
| Hypothyroidism | 290 | 137.62 | 41.32 | 0.4834 | 143.34 | 46.15 | 0.712 | 119.90 | 37.48 | 0.3639 |
| BMI>30 | 281 | 131.10 | 42.24 | 0.06543 | 142.23 | 48.61 | 0.4693 | 118.31 | 42.34 | 0.8531 |
| Gestational diabetes | 203 | 134.46 | 42.19 | 0.6404 | 147.18 | 50.61 | 0.4363 | 120.60 | 48.74 | 0.4259 |
| High risk for preterm labor | 200 | 133.76 | 38.13 | 0.4447 | 143.50 | 44.01 | 0.7848 | 117.84 | 37.93 | 0.9996 |
| Twins and multiples | 143 | 141.14 | 45.62 | 0.1742 | 141.00 | 40.23 | 0.3262 | 116.65 | 45.52 | 0.7577 |
| Pre-gestational Diabetes mellitus | 127 | 136.07 | 40.41 | 0.9569 | 145.73 | 39.36 | 0.7003 | 125.28 | 40.12 | 0.03988 |
| Hyperthyroidism | 112 | 149.05 | 55.19 | 0.01337 | 145.82 | 46.25 | 0.7428 | 126.83 | 46.33 | 0.0437 |
| Chronic hypertension | 75 | 140.21 | 46.67 | 0.4258 | 140.78 | 38.72 | 0.4282 | 125.71 | 48.71 | 0.1673 |
| Inherited thrombophilia | 71 | 134.31 | 47.17 | 0.7823 | 135.05 | 50.39 | 0.1254 | 114.99 | 43.09 | 0.5821 |
| Suspected placental abruption | 66 | 133.12 | 44.77 | 0.6212 | 148.82 | 46.84 | 0.4456 | 119.13 | 40.96 | 0.7997 |
| Intrauterine growth restriction | 65 | 126.55 | 41.39 | 0.07576 | 141.24 | 48.59 | 0.6095 | 108.59 | 35.39 | 0.04028 |
| Hypercholesterolemia | 54 | 145.99 | 43.06 | 0.09126 | 152.50 | 41.00 | 0.1568 | 128.19 | 39.27 | 0.05911 |
| Oligohydramnios | 51 | 133.41 | 41.67 | 0.6758 | 143.56 | 46.29 | 0.9016 | 110.75 | 36.75 | 0.1811 |
| Recurrent pregnancy loss | 50 | 138.01 | 52.41 | 0.775 | 139.00 | 56.91 | 0.5087 | 120.00 | 44.23 | 0.7319 |
| Fibroids | 47 | 151.60 | 42.07 | 0.014 | 158.88 | 47.41 | 0.04198 | 137.40 | 49.60 | 0.009713 |
| Impaired glucose tolerance | 47 | 133.68 | 48.64 | 0.7597 | 137.96 | 44.95 | 0.3356 | 116.73 | 42.56 | 0.8605 |
| Inflammatory bowel disease | 44 | 165.02 | 58.05 | 0.001811 | 168.06 | 56.95 | 0.008581 | 142.85 | 53.10 | 0.003231 |
| Polyhydramnios | 42 | 139.91 | 44.16 | 0.558 | 152.34 | 49.98 | 0.3085 | 120.90 | 48.52 | 0.6854 |
| Placenta previa | 39 | 158.26 | 60.84 | 0.02737 | 168.72 | 77.23 | 0.05646 | 138.38 | 79.35 | 0.1144 |
| Endometriosis | 30 | 143.88 | 30.55 | 0.1634 | 135.02 | 29.34 | 0.09335 | 113.46 | 31.23 | 0.451 |
| Epilepsy | 29 | 143.37 | 49.74 | 0.4247 | 145.20 | 54.85 | 0.9356 | 126.21 | 52.98 | 0.4025 |
| Pregnancy related hypertension | 27 | 136.21 | 35.20 | 0.9602 | 165.91 | 50.42 | 0.03562 | 123.19 | 33.84 | 0.4202 |
| Single umbilical artery | 20 | 136.64 | 48.53 | 0.9441 | 134.75 | 39.02 | 0.2851 | 121.98 | 47.11 | 0.6988 |
| Rheumatic disease | 18 | 146.19 | 41.37 | 0.3052 | 139.09 | 41.87 | 0.6005 | 129.66 | 48.69 | 0.3178 |
| BMI<18 | 17 | 135.57 | 55.50 | 0.9825 | 148.84 | 52.80 | 0.7395 | 115.68 | 27.59 | 0.752 |
| Acquired thrombophilia | 13 | 133.55 | 41.83 | 0.8448 | 143.11 | 58.17 | 0.9392 | 122.77 | 55.18 | 0.7526 |
| Uterine Mullerian anomaly | 11 | 122.22 | 28.30 | 0.1411 | 152.21 | 47.87 | 0.5991 | 120.20 | 46.32 | 0.869 |
| Cholestasis of pregnancy | 6 | 139.07 | 29.73 | 0.8027 | 186.27 | 47.95 | 0.1224 | 158.59 | 68.37 | 0.2041 |
| Asthma | 2 | 213.78 | 34.58 | 0.1934 | 235.38 | 45.69 | 0.217 | 173.69 | 25.55 | 0.1989 |

NLR

| **Diagnosis** | **Number of patients** | **Mean Trimester 1** | **SD^a^** | **P-value** | **Mean Trimester 2** | **SD^a^** | **P-value** | **Mean Trimester 3** | **SD^a^** | **P-value** |
| --- | --- | --- | --- | --- | --- | --- | --- | --- | --- | --- |
| Hyperemesis gravidarum | 413 | 2.66 | 1.02 | 0.2547 | 4.20 | 1.48 | 0.03269 | 3.54 | 1.22 | 0.3646 |
| Hypothyroidism | 290 | 2.59 | 0.98 | 0.836 | 3.85 | 1.28 | 0.01501 | 3.43 | 1.14 | 0.4882 |
| BMI>30 | 281 | 2.54 | 0.92 | 0.239 | 3.96 | 1.31 | 0.2897 | 3.36 | 1.10 | 0.07623 |
| Gestational diabetes | 203 | 2.75 | 1.05 | 0.04299 | 4.44 | 1.59 | < 0.001 | 3.54 | 1.28 | 0.4839 |
| High risk for preterm labor | 200 | 2.53 | 1.08 | 0.3612 | 3.86 | 1.13 | 0.02609 | 3.43 | 1.11 | 0.5244 |
| Twins and multiples | 143 | 2.97 | 1.05 | < 0.001 | 4.18 | 1.26 | 0.2114 | 3.48 | 1.15 | 0.9658 |
| Pre-gestational Diabetes mellitus | 127 | 2.60 | 1.03 | 0.9867 | 4.12 | 1.36 | 0.5022 | 3.48 | 1.20 | 0.9777 |
| Hyperthyroidism | 112 | 2.85 | 1.15 | 0.02532 | 4.01 | 1.22 | 0.7796 | 3.62 | 1.19 | 0.2054 |
| Chronic hypertension | 75 | 2.68 | 0.86 | 0.429 | 4.02 | 1.16 | 0.8453 | 3.56 | 1.17 | 0.5698 |
| Inherited thrombophilia | 71 | 2.49 | 0.96 | 0.3351 | 3.53 | 1.16 | < 0.001 | 3.31 | 1.33 | 0.273 |
| Suspected placental abruption | 66 | 2.64 | 1.01 | 0.7654 | 4.16 | 1.35 | 0.4973 | 3.57 | 1.26 | 0.5599 |
| Intrauterine growth restriction | 65 | 2.46 | 1.02 | 0.2794 | 4.10 | 1.46 | 0.7355 | 3.32 | 1.12 | 0.2616 |
| Hypercholesterolemia | 54 | 2.83 | 0.97 | 0.09256 | 4.07 | 1.09 | 0.8724 | 3.86 | 1.33 | 0.04241 |
| Oligohydramnios | 51 | 2.52 | 1.25 | 0.6363 | 3.84 | 1.36 | 0.3066 | 2.98 | 1.00 | < 0.001 |
| Recurrent pregnancy loss | 50 | 2.61 | 0.87 | 0.9272 | 3.69 | 1.52 | 0.1081 | 3.23 | 0.96 | 0.06623 |
| Fibroids | 47 | 3.18 | 1.35 | 0.005289 | 4.38 | 1.49 | 0.1284 | 3.95 | 1.41 | 0.0289 |
| Impaired glucose tolerance | 47 | 2.71 | 1.04 | 0.4598 | 3.81 | 1.46 | 0.2871 | 3.48 | 1.27 | 0.9803 |
| Inflammatory bowel disease | 44 | 2.96 | 1.12 | 0.04081 | 4.39 | 1.51 | 0.1336 | 3.71 | 1.23 | 0.2256 |
| Polyhydramnios | 42 | 2.83 | 1.09 | 0.1797 | 4.19 | 1.28 | 0.4745 | 3.63 | 1.46 | 0.5248 |
| Placenta previa | 39 | 2.67 | 1.11 | 0.6981 | 4.17 | 1.64 | 0.6191 | 3.65 | 1.60 | 0.5026 |
| Endometriosis | 30 | 2.49 | 0.87 | 0.4909 | 3.86 | 1.12 | 0.3919 | 3.31 | 0.92 | 0.3227 |
| Epilepsy | 29 | 2.68 | 0.95 | 0.6644 | 3.89 | 1.19 | 0.4906 | 3.61 | 1.44 | 0.6362 |
| Pregnancy-related hypertension | 27 | 2.37 | 0.69 | 0.09302 | 4.29 | 1.02 | 0.2262 | 3.58 | 0.82 | 0.5466 |
| Single umbilical artery | 20 | 2.61 | 0.81 | 0.9558 | 3.91 | 1.22 | 0.6419 | 3.55 | 1.18 | 0.7914 |
| Rheumatic disease | 18 | 2.49 | 1.00 | 0.6517 | 3.52 | 1.28 | 0.1008 | 3.48 | 1.24 | 0.986 |
| BMI<18 | 17 | 2.64 | 0.99 | 0.885 | 4.41 | 1.95 | 0.4665 | 3.35 | 1.01 | 0.6043 |
| Acquired thrombophilia | 13 | 2.62 | 0.91 | 0.9499 | 3.92 | 1.17 | 0.711 | 3.51 | 1.07 | 0.9172 |
| Uterine Mullerian anomaly | 11 | 2.25 | 0.87 | 0.2075 | 4.00 | 1.53 | 0.9211 | 3.15 | 1.40 | 0.457 |
| Cholestasis of pregnancy | 6 | 2.60 | 1.03 | 0.9955 | 5.24 | 1.54 | 0.1592 | 3.51 | 0.97 | 0.9519 |
| Asthma | 2 | 2.84 | 0.72 | 0.7183 | 4.77 | 2.94 | 0.7857 | 4.26 | 0.55 | 0.2921 |

^a^SD=standard deviation
