# Supplementary material for: Neutrophils to lymphocytes ratio and platelets to lymphocytes ratio in pregnancy: A population study
Source: PLoS One. 2018 May 22;13(5):e0196706. doi: 10.1371/journal.pone.0196706 (PMC5963784; doi:10.1371/journal.pone.0196706)
Supplement: S5 Table — (DOCX) [file pone.0196706.s008.docx]

S11. Table. Mean PLR and NLR by socioeconomic class

| **Socio-Economic Class** | **Mean trimester 1** | **SD**^a^ | **Mean trimester 2** | **SD**^a^ | **Mean trimester 3** | **SD**^a^ |
| --- | --- | --- | --- | --- | --- | --- |
| **PLR** | | | | | | |
| Low | 138.31 | 45.85 | 143.72 | 48.10 | 121.85 | 46.34 |
| Medium | 136.55 | 42.86 | 144.20 | 43.90 | 117.76 | 41.23 |
| High | 135.61 | 42.18 | 145.40 | 46.97 | 117.01 | 41.39 |
| **NLR** | | | | | | |
| Low | 2.55 | 0.98 | 3.85 | 1.30 | 3.37 | 1.14 |
| Medium | 2.63 | 0.99 | 4.04 | 1.34 | 3.50 | 1.22 |
| High | 2.65 | 0.97 | 4.15 | 1.44 | 3.55 | 1.20 |

## ^a^SD=standard deviation
