# Supplementary material for: Neutrophils to lymphocytes ratio and platelets to lymphocytes ratio in pregnancy: A population study
Source: PLoS One. 2018 May 22;13(5):e0196706. doi: 10.1371/journal.pone.0196706 (PMC5963784; doi:10.1371/journal.pone.0196706)

S9 Fig. Hemoglobin age plots: mean parameters by age and trimester (error bars represent a single standard deviation from the mean).


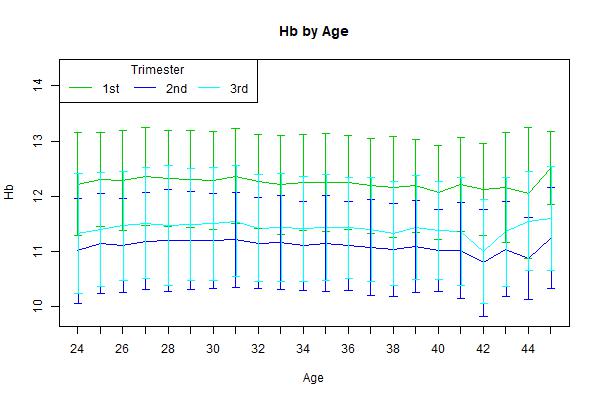

Supplement: S3 Fig — Mean parameters by age and trimester (error bars represent a single standard deviation from the mean). (DOCX) [file pone.0196706.s011.docx]
